# Colored Deacon’s Prayer

**Published:** 1872-04

**Authors:** 


THE Jacksonville (Florida) *Union* gets off this religious joke on one of its colored brethren who attempts to pray. The good colored deacon was praying for the recovery of a sick sister, and ended this way : "Oh ! Lord, help her. O ! Lord, make her well. Oh ! Lord, if you can't make her well, then, oh ! Lord, help her to grin and bear it !"

